# Synthesis, micellar structures and emission mechanisms of an AIE and DDED-featured fluorescent pH- and thermo-meter

**DOI:** 10.1039/d0ra01000f

**Published:** 2020-06-19

**Authors:** He Guo, Xiaomeng Cheng, Hongping Li, Jun Li, Jinjin Wei, Chongyang Feng

**Affiliations:** Green Catalysis Center, College of Chemistry, Zhengzhou University Zhengzhou Henan 450001 China lihongping@zzu.edu.cn +86 371 67781205 +86 371 67781205; Beijing National Laboratory for Molecular Sciences, CAS Key Laboratory of Colloid and Interface and Thermodynamics, Institute of Chemistry, Chinese Academy of Sciences Beijing 100190 P. R. China; School of Chemistry and Chemical Engineering, University of Chinese Academy of Sciences Beijing 100049 China

## Abstract

A new nanoprobe, the luminescent diblock copolymer PNIPAM(MAh-4)-*b*-P4VP (PN4P), with pH- and thermo-responsive deprotonation-driven emission decay (DDED) and aggregation-induced emission (AIE) features was designed and synthesized. The nanoprobe PN4P can form micellar structures in water with reversible dual-responsive fluorescence (FL) behavior within a wide pH range of 2–11. The critical solution temperature was found at about 32, 30 and 27 °C as the pH switched from 2, 7 to 11. The critical pH value of the probe was about 4.0, and the micelles showed a core–shell inversion in response to pH and thermal stimuli, accompanied by a desirable emission tunability. P4VP as the micellar shell at pH = 2 was more easily dehydrated with the increase in temperature as compared to PNIPAM as the micellar shell at pH > 4. The strongest dehydration of the P4VP shell would make PN4P the most strongly aggregated and the most AIE-active, which supports the 2.10-fold most distinguished thermal-responsive emission enhancement at pH = 2. Moreover, a dramatic acidochromic redshift of the emission band from 450 (pH > 4) to 490 nm (pH = 2) was observed, and the maximum emission at pH = 2 was enhanced by about 2.07-fold as compared with that at pH = 7. Therefore, the probe displays the desired dual responses and good reversibility. AIE and DDED are the two major mechanisms responsible for the dual-responsive emission change, with AIE playing a more important role than DDED. This work offers a promising approach to interpreting temperature (range from 28 to 40 °C) and pH changes (range from 2 to 7) in water.

## Abstract 2

A nanoprobe in water features pH- and thermal-responsive micellar/clustering structures, deprotonation-driven emission decay (DDED) and aggregation-induced emission (AIE).

## Graphical abstract

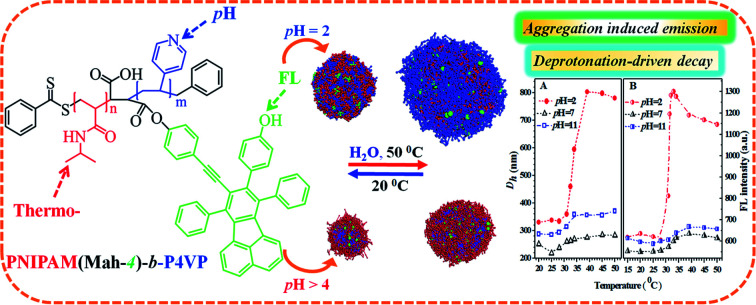

## 1. Introduction

Stimuli-responsive polymers are powerful tools in drug delivery,^1–4^ tissue engineering,^5^ biological sensors,^6–8^ and fluorescence (FL) probes.^9–11^ In recent years, many stimuli-responsive polymers, whose properties change in response to ionic strength, light, pH and temperature, have been studied,^12^ and molecular level solubility changes of segments could be triggered by such stimuli as temperature,^13^ pH,^14^ CO_2_ (ref. 15 and 16) and electric/magnetic field.^17^ Among the diverse stimuli, thermal- and pH-responsive polymers have been widely explored due to their potential *in vivo* applications,^11,18^ and polymers with both thermal and pH stimuli are extensively studied dual responsive systems.^19–23^ As is known, poly(*N*-isopropylacrylamide) (PNIPAM) is a thermal-responsive polymer with a lower critical solution temperature (LCST) of about 32 °C.^24^ Owing to its thermo-sensitivity and desired biocompatibility, PNIPAM-containing materials have been extensively employed in temperature-targeted therapy systems and drug delivery materials.^18,25^PNIPAM was also used to regulate the fluorophore emission *via* thermally-induced aggregations and micro-environmental changes.^26,27^ Polymers with end-capped dye moieties^26^ or at the junction between two blocks have been reported.^27^ Guo *et al.*^26^ synthesized a fluorescent nano-thermometer PNIPAm-MAh-4 with aggregation-induced emission (AIE) character, which showed enhanced emission in water with a temperature increase and the maximum emission at 50 °C was about 7-fold stronger than that at 15 °C. In contrast, a carbazole-containing PNIPAM copolymer solution^27^ exhibited 22 times emission enhancement along with a temperature drop from 50 to 25 °C. Besides, a few double-hydrophilic diblock copolymers with both thermo and pH responses were reported, together with a core–shell inversion,^23^ which included poly(*N*-isopropylacrylamide)-*block*-poly(4-vinylpyridine) (PNIPAM-*b*-P4VP), PNIPAM-*b*-PAA and PPO-*b*-PDEA. Among these, PPO-*b*-PDEA micelles were unstable at ambient temperature and the PNIPAM-*b*-PAA counterpart had strong hydrogen bonds between PAA and PNIPAM blocks, which greatly limited their further applications.^23^PNIPAM-*b*-P4VP seemed to be the most desirable dual-responsive copolymer because there were no strong hydrogen bonds between P4VP and PNIPAM blocks, and PNIPAM covers a critical aggregation temperature range that is especially suitable for biomedical applications.

In addition, amphiphilic block copolymers can form micelles *via* self-assembly in selective solvents, and various dye-functionalized amphiphilic block copolymers have been prepared to realize further FL property control through the regulation of their self-assembled nanostructures in solutions.^27–32^ Cheng *et al.*^28,31^ reported their work on the structure and emission behavior tuning of self-assembled fluorescent composites (SAFC) between dye molecule and PS-*b*-P4VP in CO_2_-expanded liquids. The morphology and emission behaviors of SAFC were considerably pressure dependent and the maximum FL intensity of SAFC at 5.41 MPa was enhanced 14-fold.^28^ Tan *et al.*^32^ designed FL vesicles *via* the complexation of FL polyoxometalate and stimuli-responsive triblock copolymer PEO-*b*-PS-*b*-PDMAEMA by electrostatic interactions, which showed on-off switchable emission behavior along with pH tuning of the reversible micelle-to-vesicle change. Unfortunately, these polymers had no temperature response and their micellization process often involved bio-incompatible organic solvents.

**Fig. 1:**
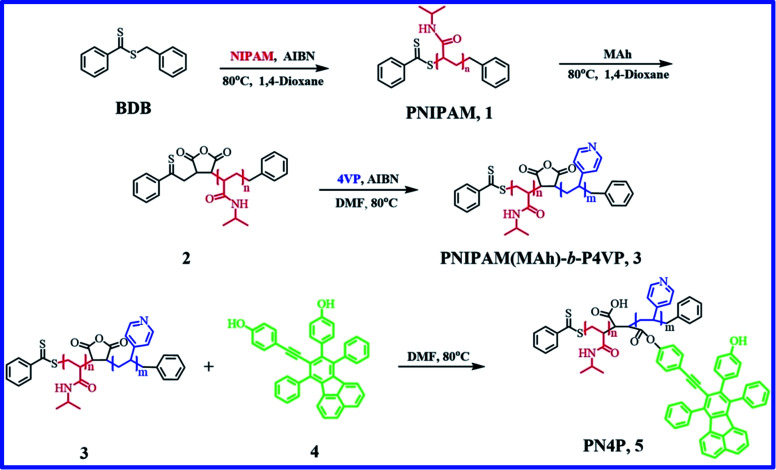
Synthetic route for PN4P (or compound 5).

To date, AIE-featuring materials have received great attention owing to their potentials in stimuli-responsive materials,^33,34^ life sciences^11,35,36^ and biomedical engineering.^4,37,38^ For example, TPE polymers^33^ were synthesized with AIE character, pH response and solvatochromic behavior, and the P1–P3 based sensors exhibited pH response through the protonation of the amino groups and could identify twelve different nitroarenes in water; however, the above TPE polymers are not temperature responsive. Very recently, AIE-active nanoprobes PNIPAM-*b*-P(DPA-*co*-TPE) and P(NIPAM-*co*-TPE)-*b*-PDPA with pH and thermo responses were reported,^11^ and the respective FL intensities were enhanced 7- and 3-fold for PNIPAM-*b*-P(DPA-*co*-TPE) and P(NIPAM-*co*-TPE)-*b*-PDPA by solution pH or temperature tuning. Although PNIPAM-*b*-P4VP is a desired thermo- and pH- dual-responsive copolymer,^23^ little attention has been paid to the luminescence performance of chromophores regulated by PNIPAM-*b*-P4VP-based polymers, and it appears that thermal and pH responsive fluorescent sensor materials remain a highly unexplored area. Therefore, our objectives include the design of a novel biocompatible dye-labeled polymer probe with pH and thermo dual-responses suitable for biomedical applications, showing entirely different pH-driven emission trends against temperature, and finding the dual-responsive micellar structures and emission mechanisms of the probe in water, uncovering the dominant factors that decide the pH- or thermal responses, as well as obtaining the sensitivity range or suitable working temperature and pH range of the probe. The innovation of this work is to ensure that the developed pH and temperature-responsive probe has the features of totally different emission mechanisms such as AIE/deprotonation-driven emission decay (DDED) against temperature within different pH ranges. Thus, a PNIPAM-*b*-P4VP-based polymer labeled with a dye unit possessing both AIE and DDED features appears to be an ideal choice. Accordingly, in this work, we focus on the synthesis and dual-responsive emission behaviors of a double-hydrophilic diblock copolymer PNIPAM(MAh-4)-*b*-P4VP (PN4P, Fig. 1). Herein, PNIPAM and P4VP blocks, as well as 4 moieties,^26^ endow the PN4P with thermal-, pH-, and FL-responsive properties. Particularly, the dye unit 4, with partially propeller-like moieties and phenolic OH groups as an archetypal AIE/DDED module, was used to label the PNIPAM-*b*-P4VP and was expected to serve as an intriguing building block for AIE/DDED-active luminogenic polymers. The micellar/clustering behavior of the probe in water was studied by solution dispersibility, DLS, NMR, and TEM measurements, and the emission performance was systematically examined by temperature and pH tuning. As expected, the PN4P probe demonstrates obvious DDED and AIE features in different microenvironments, and simultaneously displays good reversibility and is more sensitive within the pH range of 2 to 7 and temperature range from 28 to 40 °C.

## 2. Experimental section

### 2.1. Materials

4-Vinyl pyridine (4VP, Alfa Aesar, Beijing, 96%) was distilled under reduced pressure and stored in a refrigerator. *N*-Isopropylacrylamide (NIPAM, Shanghai Chemical Reagents Co., 98%) was recrystallized from toluene and *n*-hexane (1 : 3). Maleic anhydride (MAh, Beijing Chemical Reagents Co., 99%) was recrystallized from anhydrous chloroform. 2,2-Azobis(isobutyronitrile) (AIBN, Beijing Chemical Reagents Co., 98%) was recrystallized from ethanol twice. 1,4-Dioxane (Tianjin Chemical Reagents Co., 99%) was distilled in the presence of sodium slices. Tetrahydrofuran (THF, Beijing Chemical Reagents Co., 99%) was distilled from sodium benzophenone ketyl under argon immediately prior to use. Magnesium powder, potassium hydroxide, iodine, MgSO_4_, CuI and PdCl_2_, dichloromethane, chloroform, ethyl acetate, methanol, ethanol, petroleum ether and *ortho*-xylene (Beijing Chemical Reagents Co., analytical pure grade) were used as received. The ethanol for absorption (Abs) was HPLC grade (≥99.9%), obtained from Aladdin, and was used as received. 4-(9-(2-(4-Hydroxyphenyl)ethynyl)-7,10-diphenylfluoranthen-8-yl)phenol (4) and benzyl dithiobenzoate (BDB) were synthesized in our lab according to a reference procedure.^26^PNIPAM (*M*_n_ = 16 200 g mol^−1^) and copolymer PNIPAM(MAh)-*b*-P4VP (*M*_n_ = 26 200 g mol^−1^) were synthesized according to a procedure described previously,^26,28^ and the detailed reaction conditions for the preparation of PNIPAM and PNIPAM(MAh)-*b*-P4VP, the number average molecular weight (*M*_n_) and polydispersity index (*M*_w_/*M*_n_) of PNIPAM and PNIPAM(MAh)-*b*-P4VP determined by gel permeation chromatography (GPC) are tabulated in Table 1. The PNIPAM(MAh-4)-*b*-P4VP (PN4P) probe was synthesized according to a reference method,^26^ the detailed reaction conditions and the number average molecular weights from the GPC measurement are tabulated in Table 2. The degree of labeling (molar ratio of 4 to NIPAM) in PN4P was determined to be 0.063% (or 1/1600, Table 2) by comparison of the absorbance with compound 4 (A390 nm) dissolved in ethanol at 25 °C. The synthesis route for PN4P is summarized in Fig. 1. The pH buffers from Sigma-Aldrich were used as received. The lower pH value of the solvent was adjusted with 0.1 mol L^−1^ HCl and the higher pH value of the solvent was adjusted with 0.1 mol L^−1^ NaOH. All other reagents were analytical grade and were used without further purification.

**Table 1:** Polymerization of PNIPAM (1) and PNIPAM(MAh)-*b*-P4VP (2) under different reaction conditions

Sample	*t* (h)	Initiator	BDB/AIBN	*T* (K)	*M*_n_ a	*M*_w_/*M*_n_^a^	Yield (%)
1	9	AIBN	7 : 1	353.2	16 200	1.16	55.6
2	24	AIBN	7 : 1	353.2	26 200	1.24	58.3

a Number-average molecular weight (*M*_n_) and polydispersity index (*M*_w_/*M*_n_) were determined by GPC in THF at 313.2 K, flow rate 1 mL min^−1^.

**Table 2:** PN4P results^a^

Sample	PNIPAM/4^b^	NIPAM/4^c^	*M*_n,GPC_ d	*M*_w_/*M*_n_^d^
PN4P	1 : 5	1600 : 1	26 000	1.34

a The reaction carried out at 80 °C for 24 h. Sample PN4P was prepared from sample 2 shown in Table 1.

b Molar feed ratio of PNIPAM to 4.

c Molar ratio of NIPAM to 4 obtained from PN4P using the absorbance calibration, by comparison of absorbance of PN4P with 4 (*A*_390 nm_) dissolved in ethanol at 25 °C.

d Determined by GPC in THF at 313.2 K, flow rate of 1 mL min^−1^.

### 2.2. Synthesis

#### 2.2.1 PNIPAM (1)

**Fig. 2:**
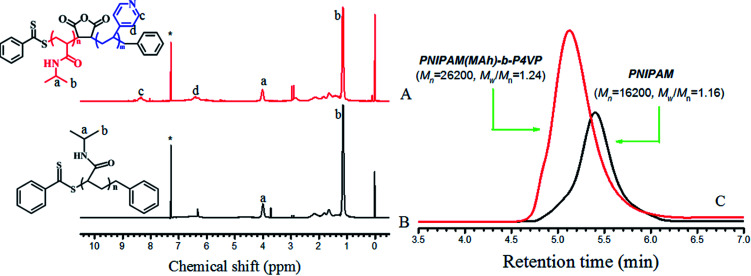
^1^H NMR spectra of (A) PNIPAM(MAh)-*b*-P4VP in CDCl_3_, (B) PNIPAM in CDCl_3_. Solvent signals are marked with asterisks (*); (C) GPC of PNIPAM and PNIPAMM(MAh)-*b*-P4VP.

PNIPAM was prepared *via* the reversible addition–fragmentation chain transfer (RAFT) polymerization method as reported previously.^26^NIPAM (2 g, 0.0176 mol), AIBN (0.002 g, 0.0122 mmol), BDB (0.0209 g, 0.0855 mmol) and 1,4-dioxane (3 mL) were added to a 20 mL Schlenk tube, followed by three freeze–vacuum–thaw cycles. The tube was sealed under vacuum and then immersed in an oil bath at 80 °C with magnetic stirring. After reaction for 9 h, the tube was cooled to room temperature and opened to the air. The polymer was dissolved in a suitable amount of 1,4-dioxane, and then precipitated by dropping the solution into anhydrous ether, followed by filtration. Repeating the procedure of dissolving–precipitation–filtration three times, the obtained pink product was dried under vacuum at 30 °C for 24 h to obtain the PNIPAM. ^1^H NMR (CDCl_3_, 400 MHz): *δ* 4.00 (s, (CH_3_)_2_C*H*NH), 1.13 (s, (C*H*_3_)_2_CHNH). The ^1^H NMR data, the number average molecular weights and polydispersity index from GPC for PNIPAM are also displayed in Fig. 2B and C, respectively.

#### 2.2.2 PNIPAM-MAh (2)

PNIPAM (1 g, 0.0625 mmol), MAh (0.28 g, 2.85 mmol), and 1,4-dioxane (4 mL) were added to a 20 mL Schlenk tube, followed by three freeze–vacuum–thaw cycles. Afterwards, the vacuum-sealed tube was immersed in an oil bath at 80 °C with magnetic stirring. After reaction for 6 h, the tube was quickly cooled to room temperature. The polymer was dissolved in a suitable amount of THF and then precipitated in anhydrous ether to remove the excess MAh, followed by filtration. The operation of dissolving–precipitation–filtration was repeated three times, and the pink polymer obtained was dried under vacuum at 30 °C for 24 h to obtain the PNIPAM-MAh. IR (KBr, cm^−1^): 1730.32 (*ν*_C=O_); 1652.72 (*ν*_NH–C=O_); 1172.53 (*ν*_C=S_).

#### 2.2.3 PNIPAM(MAh)-*b*-P4VP (3)

4VP (0.19 g, 4.66 mmol), PNIPAM-MAh (0.35 g, 0.0218 mmol), AIBN (0.0037 g, 0.0225 mmol), and DMF (3 mL) were added to a 20 mL Schlenk tube with a magnetic bar. After three freeze–vacuum–thaw cycles, the vacuum-sealed tube was immersed in an oil bath at 80 °C and reacted for 24 h. The tube was cooled to room temperature and opened to the air. The polymer was dissolved in DMF, and then precipitated by adding the solution into anhydrous ether dropwise, followed by filtration. The process of dissolving–precipitation–filtration was repeated three times and the obtained pale-yellow product PNIPAM(MAh)-*b*-P4VP was dried under vacuum at 30 °C for 24 h. ^1^H NMR (CDCl_3_, 400 MHz): *δ* 8.0–8.2 (m, 2H), 6.0–6.3 (m, 2H). The ^1^H NMR and GPC data for PNIPAM(MAh)-*b*-P4VP can be found in Fig. 2A and C, respectively.

#### 2.2.4 4-(9-(2-(4-Hydroxyphenyl)ethynyl)-7,10-diphenylfluoranthen-8-yl)phenol (4)

Compound 4 was previously prepared in our lab according to the method reported.^26^^1^H NMR, *δ* (CD_3_SOCD_3_, ppm): 9.84 (s, –OH), 9.30 (s, –OH), 6.30–7.85 (aromatic proton).

#### 2.2.5. PNIPAM (MAh-4)-*b*-P4VP (5 orPN4P)

In a typical polymerization procedure, PNIPAM(MAh)-*b*-P4VP (0.500 g, 0.0192 mmol), compound 4 (0.050 g, 0.0889 mmol) and DMF (3 mL) were successively added to a dry Schlenk tube, followed by three freeze–vacuum–thaw cycles. Afterwards, the vacuum-sealed tube was immersed in an oil bath at 80 °C with magnetic stirring. After reaction for 24 h, the tube was promptly cooled to room temperature and opened to the air. The polymer was dissolved in the appropriate amount of DMF, and then precipitated in anhydrous ether. The dissolving–precipitation–filtration process was repeated three times and the resultant polymers (PN4P or 5) were dried under vacuum for 24 h to finally obtain the pale-yellow product. The number average molecular weights and polydispersity index from GPC for PN4P, and the labeling degree of the fluorescent moiety 4 in PN4P (molar ratio of NIPAM to 4) from the absorbance measurement can be found in Table 2. However, considering the low labeling degree of 4 in PN4P (molar ratio of NIPAM/4 = 1600/1), it was somewhat difficult to confirm PN4P by IR or NMR data; we then turned to a thin-layer chromatography (TLC) test to help to identify the PN4P. When comparing PN4P and 4 under the same chromatography conditions using a mixture of ethyl acetate and petroleum ether (3 : 1, volume ratio), we observed two blue-green light-emitting spots at different positions on the same TLC plate under the illumination of a UV lamp, with *R*_f_ value of about 7.0 and 0.0 mm for 4 and PN4P, respectively. This proved that PN4P was prepared successfully and demonstrates that PN4P is more polar than 4.

### 2.3 General information

Fourier transform infrared (FT-IR) spectra were recorded on a NEXUS-470 spectrometer. ^1^H NMR spectra were obtained from a DRX-400 NMR instrument with tetramethylsilane as an internal standard. The average molecular weights (*M*_w_ and *M*_n_) and polydispersity indexes (PDIs) were measured by an Agilent 1200 Series LC Gel permeation chromatography instrument equipped with a G1310A HPLC Iso Pump, G1362A differential refractive index (RI) detector and four columns at 40 °C, using monodispersed polystyrene (PS) as a calibration standard. THF was used as the eluent at a flow rate of 1.0 mL min^−1^. UV-vis spectra were taken on a Persee TU-1901 spectrophotometer with a temperature controller. The pH- and thermal-responsive fluorescence measurements were carried out on a Hitachi FL-4600 spectrofluorometer. The emission spectra were recorded at an excitation wavelength of 390 nm. The micellar/clustering morphology of PN4P in water was characterized by transmission electron microscopy (TEM), using a H-800 microscope (Hitachi, Japan). TEM samples were prepared by dropping sample solutions on carbon-coated copper grids, absorbing the solvent on filter paper, and evaporating the solvent at room temperature. Dynamic light scattering (DLS) measurements were carried out to analyze the hydrated diameter of PN4P micelles in aqueous solution with a Nano Plus-3 Particle Size analyzer (Micromeritics Instruments, USA), equipped with a He–Ne laser (664.4 nm, 70 mW). The Nano Plus version 5.22 software package (Micromeritics Instrument) was used to assist the measurements and data analysis. The hydrodynamic radius (*R*_h_) can be extracted from the Stokes–Einstein equation as follows:

1 *R*_h_ = *k*_B_*T*/6π*ηD*,

where *T* denotes the absolute temperature, and the parameters *k*_B_, *D* and *η* represent the Boltzmann constant, the diffusion coefficient and the solvent viscosity, respectively. All the pH measurements were made with a PHS-2F precision pH meter (Inesa Instruments of Shanghai, China), which was calibrated with standard buffer solutions (pH 6.86 and pH 9.18).

## 3. Results and discussion

### 3.1 The pH- and thermal-responsive aggregation/micellization of PN4P in water

In this section, the combination of DLS, ^1^H NMR, TEM and dispersibility data against temperature/pH were used to help clarify the clustering structure of PN4P in water.

**Fig. 3:**
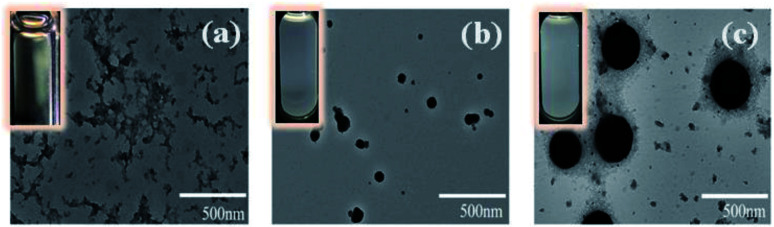
(a–c) Temperature-responsive TEM images of PN4P in water at pH = 2 and the concentration of 0.5 mg mL^−1^ at (a) 20 °C, (b) 34 °C, (c) 50 °C. Inset: the corresponding dispersibility images of the solution.

**Fig. 4:**
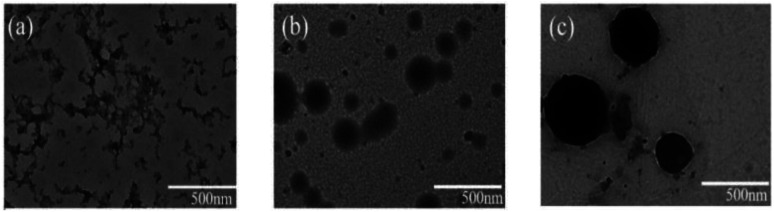
pH-responsive TEM images of PN4P in water at 20 °C and the concentration of 0.5 mg mL^−1^ at (a) pH = 2, (b) pH = 7, (c) pH = 11.

**Fig. 5:**
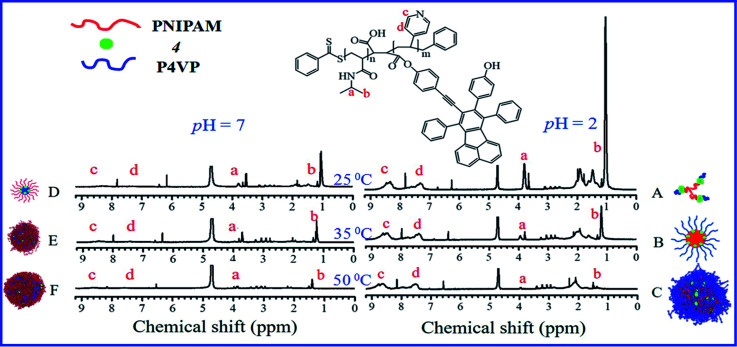
^1^H NMR spectra obtained for PN4P in D_2_O at (A) 25 °C and pH = 2, (B) 35 °C and pH = 2, (C) 50 °C and pH = 2, (D) 25 °C and pH = 7, (E) 35 °C and pH = 7, (F) 50 °C and pH = 7.

The thermal-responsive solubility/dispersibility and TEM images of PN4P aqueous solutions at pH = 2 are displayed in Fig. 3, and the pH-responsive TEM images at 20 °C are shown in Fig. 4. It was found from the TEM data that the structures of the copolymer clusters appeared as spherical composite micelles under conditions of pH = 2 and temperature ≥34 °C (Fig. 3), or at conditions of 20 °C and pH > 2 (Fig. 4). Firstly, it was found that the dispersibility state of the PN4P aqueous solutions at pH = 2 (inset of Fig. 3) changed from the transparent state (Fig. 3a, 20 °C) to an emulsion (Fig. 3b, 34 °C), and then to a turbid state with perceptible precipitation (Fig. 3c, 50 °C), which corresponds to the worsening solubility of the copolymer as temperature increased. In other words, the clustering degree of copolymer was enhanced with increasing temperature, as reflected in the micelle morphology shown in the TEM images (Fig. 3). Although the structures of the copolymer cluster exhibited core-shell-like spherical composite micelles (Fig. 3b and c), more NMR data are still needed to verify which block would be the core or shell-forming segment. Fig. 5 shows the ^1^H NMR spectra of PN4P in D_2_O at different temperatures and pH values. The characteristic peaks of the methine and methyl protons of the PNIPAM block are supposed to be at about 3.8–4.0 (peak a) and 1.0–1.4 ppm (peak b), and the chemical shifts at about 8.4–8.6 (peak c) and 7.3–7.5 ppm (peak d) can be assigned to the 2,2′ and 3,3′ protons of the pyridine ring of the P4VP block. At pH 2 and 25 °C, it is apparent that the copolymer was molecularly dissolved since all the featured signals belonging to both blocks are visible (Fig. 5A). When temperature increased to 35 °C at pH 2 (Fig. 5B), the signals of the methyl and methine protons of PNIPAM block at about 1.2 and 3.9 ppm were substantially suppressed, indicating that the PNIPAM blocks may form the less mobile and sparsely aggregated micellar cores. However, as temperature was further increased to 50 °C at pH 2 (Fig. 5C), the signals of the characteristic peaks of the PNIPAM block at about 1.4 and 3.9 ppm were greatly restrained, signifying the formation of the least mobile and densely aggregated PNIPAM micellar cores. In contrast, as the pH value is increased to 7 at varied temperatures (Fig. 5D–F), the signals attributed to the 2,2′ and 3,3′ protons of the pyridine ring of the P4VP block at about 8.5 and 7.4 ppm almost disappeared, suggesting the poorer mobility and worse solvation of P4VP blocks, and therefore the P4VP blocks preferred to form micellar cores as the pH value increased. Meanwhile at pH = 7, we also found that the signals of the featured peaks of PNIPAM block at about 1.0–1.4 and 3.8 ppm were becoming more and more weak with temperature increase, which may suggest that the shell-forming PNIPAM blocks were getting more and more densely aggregated as temperature increased at pH = 7. We can infer from the combined NMR and TEM data that the copolymer could form core–shell micelles/clusters with P4VP cores and PNIPAM shells at pH = 7 (Fig. 5D–F), or micelles with PNIPAM cores and P4VP shells (Fig. 5B and C) of larger size (Fig. 3c) at 50 °C and pH = 2, or smaller-sized aggregates (Fig. 3a) at pH = 2 and 20 °C. Regardless of the pH value, the micelles/clusters became more and more aggregated and the size of the PN4P cluster became larger with increasing temperature, which will be proved later by the hydrated diameter data from the DLS in Section 3.2.3.

### 3.2 The pH- and thermal-responsive properties and plausible emission mechanism of PN4P in water

**Fig. 6:**
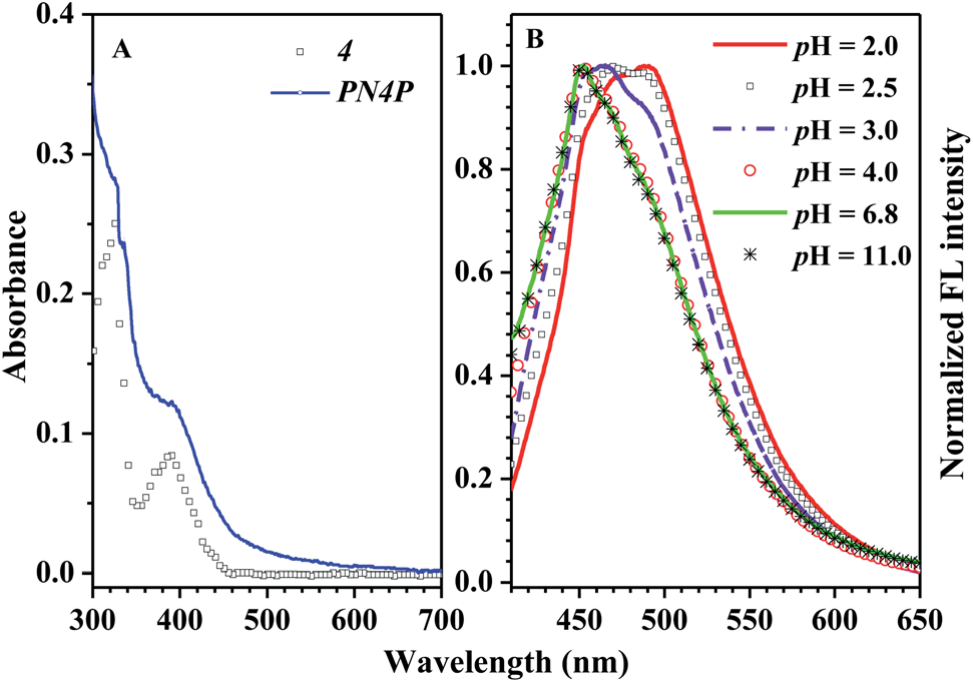
(A) Absorbance spectra of PN4P and 4 at 25 °C. 4 in ethanol (squares) at the concentration of 1 × 10^−5^ mol L^−1^, and PN4P in water at pH = 2 (solid line) with concentration of 1.0 mg mL^−1^. (B) Normalized FL spectra against pH value at 25 °C, PN4P in water with concentration of 0.5 mg mL^−1^ and *λ*_ex_ = 390 nm.

**Fig. 7:**
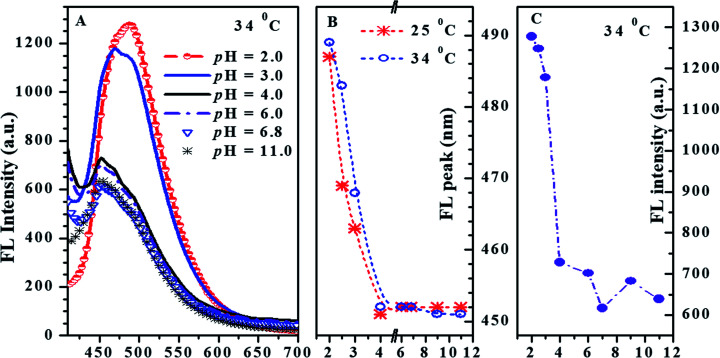
(A) Emission spectra of PN4P at varied pH and 34 °C; the effect of pH on the emission peak wavelength (B) and maximum FL intensity (C) of PN4P at constant temperature. PN4P in water at the concentration of 0.5 mg mL^−1^ and *λ*_ex_ = 390 nm.

**Fig. 8:**
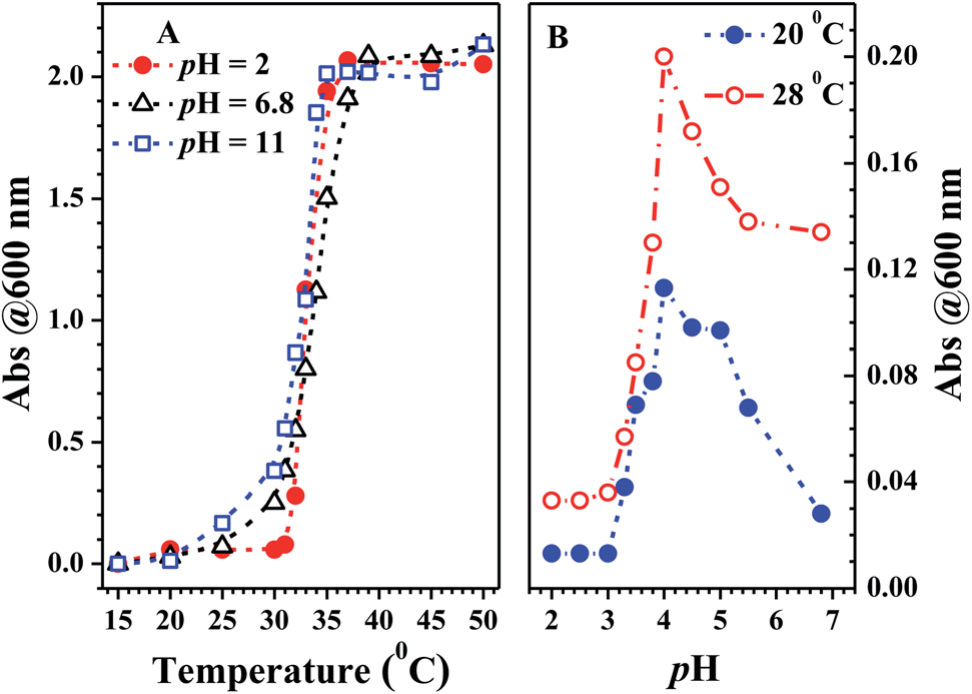
Solution turbidity of PN4P in water. (A) Temperature responses at pH = 2, 6.8 and 11; (B) pH responses at 20 and 28 °C. PN4P in water at the concentration of 0.5 mg mL^−1^, and turbidity data measured as a function of absorbance (Abs) at 600 nm.

The absorption and fluorescence of PN4P in water within a wide pH range (pH = 2–11) were measured, and the results are displayed in Fig. 6–8.

The absorbance spectra of PN4P and 4 at 25 °C can be found in Fig. 6A. The normalized pH-responsive emission spectra of PN4P in water at 25 °C are shown in Fig. 6B. A dramatic redshift of the emission band from 451 (pH > 4) to 487 nm (pH = 2) at 25 °C was observed in aqueous solution of the probe (Fig. 6B). In contrast, Fig. 7 displays the emission spectra at varied pH at 34 °C, the pH-dependent emission intensity and peak wavelength of PN4P in water. It is interesting that the pH-responsive emission peaks at 34 °C (Fig. 7B) actually tell a similar story to that for 25 °C, with a peak redshift from 451 (pH > 4) to 489 nm (at pH = 2) being recorded.

#### 3.2.1 The pH-responsive emission mechanism of PN4P in water

As shown in Fig. 7A and B, PN4P exhibits a maximum emission at about 451 nm in water within the pH range of 4–11, and there was almost no peak-shift observed with the decrease in pH until pH = 4. Then, the addition of more acid into the aqueous solution resulted in enhanced emission, along with the respective new emission maxima at 468 (pH = 3) and 489 nm (pH = 2). Simultaneously, we also found that the emission intensity of PN4P at 34 °C decreased monotonically with the increase in pH until pH = 6.8, along with a sharp FL attenuation within the pH range of 2–4, and the maximum FL intensity at pH = 2 was enhanced by about 2.1-fold as compared with that at pH = 6.8 (Fig. 7C). One possible reason for the pH-dependent emission could be because it is reasonable to think about a deprotonated state being involved since the dye unit 4 of PN4P has a free phenolic OH group and water is able to support the charges. The emission enhancement along with the peak-shift could be attributed to a pH-driven switching of the deprotonation equilibrium as follows. If PN4P-n represents the neutral form of PN4P (the chromophore group 4 with the phenol unit), and PN4P-d denotes the deprotonated form (fluorophore group 4 with the phenoxide ion unit), the phenol unit of group 4 might offer its hydrogen ion and hydrogen-bond with water at conditions of pH > 4, and the emission peak at 451 nm could be proof of the formation of PN4P-d, whereas the peak at around 489 nm is allocated to the neutral form PN4P-n. Obviously, the formation of PN4P-d, the weaker fluorescent form, would induce an emission decay accompanied by a blue peak-shift (Fig. 7). The deprotonation equilibrium of PN4P was introduced to explain the spectra change, and the pH-responsive deprotonation equilibrium of the phenolic OH groups was anticipated to influence the absorption and emission spectra of PN4P. As expected, the data in Fig. 6B and 7B (25 °C) help to support the above assumption of deprotonation equilibrium. At conditions of pH < 4, mainly PN4P-n, the stronger fluorescent form exists, with an emission peak at about 488 nm, whereas at conditions of pH > 4, PN4P-d, the weaker fluorescent form occurs with emission peak at about 451 nm; *i.e.*, both PN4P-n and PN4P-d will coexist within the pH range of 4–11. In other words, the increase in solution basicity might shift the deprotonation equilibrium towards a greater amount of PN4P-d, together with a decay in emission intensity within the pH range of 2–7 (Fig. 7C), which is in favor of the deprotonation-driven emission decay mechanism.

#### 3.2.2 The critical pH and critical solution temperature (CST) of the micellization of PN4P in water

The pH responses of emission peak wavelength and solution turbidity (absorbance at 600 nm) of the copolymer PN4P in water at constant temperature are shown in Fig. 7B and 8B, respectively. The transition of the solution turbidity against pH (Fig. 8B) suggests a critical aggregation pH value at about 4.0, highly likely due to the deprotonation of the P4VP block as mentioned by Xu *et al.*^23^ The effect of pH on the emission peak-shift at constant temperature (Fig. 7B) also supports a critical clustering pH value of around 4.0. The probe could form core–shell micelles in water at a certain critical temperature with a core–shell inversion at pH = 4. In other words, at the critical temperature, PN4P in water could self-assemble into core–shell micelles, together with a micellar structure inversion from PNIPAM cores and P4VP shells at pH < 4 to PNIPAM shells at pH > 4. As such, the next important task was to find the critical temperature of micellization.

From Fig. 8A, the critical solution temperature (CST) of PN4P was determined to be at about 32, 30 and 27 °C as the pH value switched from 2, 7 to 11, owing to the worsening solubility of the shell-forming block with increasing pH and temperature. Obviously, the solubility improvement in PN4P would lead to a rise in the CST.

In brief, the above data obtained from solution turbidity (Fig. 8A) and emission peak shifts (Fig. 7B) help suggest a CST around 32 °C in acid water and a critical pH value around 4.0, corresponding to a micellar core–shell inversion for PN4P. Therefore, from the combined NMR and TEM data, we can speculate that the copolymer could form core–shell micelles with a P4VP core at pH > 4.0 (Fig. 5D–F), or growing-sized micelles with a PNIPAM core (Fig. 5B and C) at conditions of pH < 4.0 and *T* > 32 °C (Fig. 3b and c) whereas smaller-sized aggregates formed at pH < 4.0 and *T* < 32 °C (Fig. 3a).

#### 3.2.3 The dual-responsive emission mechanisms of PN4P in water

**Fig. 9:**
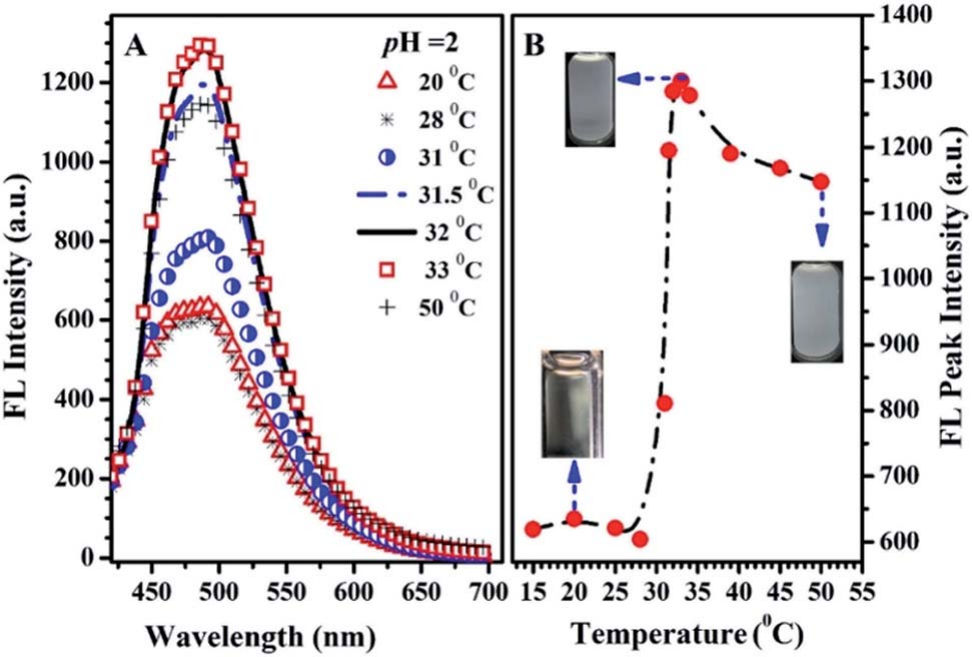
The emission spectra (A) at various temperatures; the thermo-responsive emission peak intensity and solution dispersibility (B) of PN4P in water at pH = 2 and concentration of 0.5 mg mL^−1^ and *λ*_ex_ = 390 nm.

**Fig. 10:**
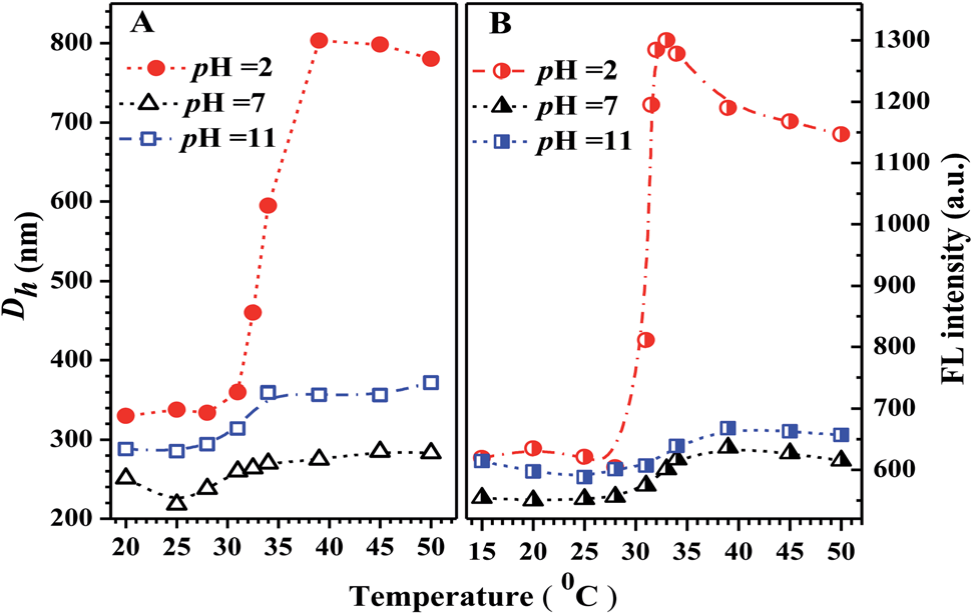
Temperature responses of (A) hydration diameter (*D*_h_) and (B) the maximum emission intensity of PN4P in water at different pH values. PN4P at the concentration of 0.5 mg mL^−1^, and *λ*_ex_ = 390 nm.

The emission spectra at various temperatures and the thermal-responsive emission peak intensity of PN4P in water at pH = 2 are shown in Fig. 9. The temperature responses of hydration diameter (*D*_h_) and emission peak intensity of PN4P in water at different pH are displayed in Fig. 10. As shown in Fig. 9, PN4P in pH = 2 water displays relatively weak emission at <31 °C with two emission peaks at 488 and 451 nm being assigned to PN4P-n and PN4P-d, respectively. With the increase in temperature above 31 °C, the emission peak at 451 nm gradually became vague while the peak at 488 nm became more dominant (Fig. 9A). As the temperature increased, firstly we saw a slight change in the FL intensity of the PN4P aqueous solution within the temperature range of 15 to 28 °C, followed by a swift emission enhancement from 29 to 33 °C and then a slow FL decay until 50 °C (Fig. 9B). Obviously, PN4P exhibited a remarkable emission enhancement between 29 and 33 °C, and the maximum FL intensity at 33 °C was enhanced 2.10-fold as compared with that at 15 °C (Fig. 9B). In contrast, although a similar thermal response of emission behavior to that at pH = 2 was observed for PN4P in aqueous solution at pH = 7 and 11, (Fig. 10B), the corresponding emission enhancement against temperature was much weaker than that at pH = 2, and the maximum FL intensity at 33 °C was only enhanced 1.08- (pH = 7) and 1.03-fold (pH = 11) compared with that at 15 °C, respectively. Furthermore, the hydration diameter (*D*_h_) of PN4P in water at different pH values also exhibited a similar thermal-responsive trend to the corresponding emission intensity (Fig. 10). For example in aqueous solution at pH = 2, a mild increase in the polymer clustering size was recorded within the temperature range of 25 to 30 °C, then a strikingly drastic size enhancement from 31 to 39 °C and a subsequent small size shrinkage until 50 °C were observed (Fig. 10A). The respective maximum clustering size *D*_h_ at 39 °C was about 800, 280 and 360 nm for PN4P at pH = 2, 7 and 11. The maximum *D*_h_ at 39 °C of PN4P in pH = 2 water was enhanced by 2.43-fold as compared with that at 20 °C, whereas the counterparts were merely increased by 1.10- (pH = 7) and 1.24-fold (pH = 11) as compared with that at 20 °C.

Why was it that *D*_h_ at pH 2 > *D*_h_ at pH 11 > *D*_h_ at pH 7 at comparable temperature (Fig. 10A)? The mechanism of the pH response of *D*_h_ could be as follows. Above the critical solution temperature (CST), the PN4P probe in water could self-assemble into core–shell micelles with P4VP shells at pH 2, but with PNIPAM shells at pH = 7 and 11. In aqueous solution at pH = 7, the minimum *D*_h_ could be due to the weakest dehydration of PN4P micelles with PNIPAM shells corresponding to the relatively strong hydrogen bonding between the acylamino N of PNIPAM and the OH group of neutral water. In aqueous solution at pH 11, basic water could not supply enough protons for the hydrogen bonding between the acylamino N of PNIPAM and water, thus the dehydration degree of PN4P micelles with PNIPAM shells became stronger, or the hydrogen bonding between PNIPAM and basic water became weaker as compared with that in neutral water, so we observed *D*_h_ at pH 11 > *D*_h_ at pH 7. The maximum *D*_h_ in acid water at pH = 2 might be due to the strongest dehydration of PN4P micelles with P4VP shells and PNIPAM cores, where the coupling between the pyridine N of the P4VP blocks and acid water would lead to the most intense degree of aggregation of the probe.

The possible reasons for the dual-responsive emission variation might be as follows: PN4P is a PNIPAM-*b*-P4VP-based polymer labeled with a dye unit 4 possessing AIE and deprotonation-driven emission decay (DDED) features under suitable conditions. Besides, PN4P can self-assemble into micellar structures in water to form core-shell-like micelles with 4 units anchored in the middle layer between the micellar cores and shells. We prefer to think that the AIE effect and the deprotonation equilibrium may be the two major factors responsible for the thermal- and pH- responsive FL changes of PN4P in water. Firstly, as shown in Table 2, the molar ratio of PNIPAM to unit 4 in PN4P used in this work is 15/1, implying that the 4 groups are likely separated from each other if polymer chains are in the hydrated coil state at lower temperatures. However, high temperature generally supports the polymer chains changing from coils to globules, which favors the aggregation of 4 and activates the restricted intra-molecular rotation (RIR) of the propeller moiety of fluorogen 4 and the resultant AIE phenomena. The thermally driven intra-polymer clustering of 4 units would possibly restrict the intra-molecular rotation in the fluorogen, which helps to enhance the emission of the 4 units. Secondly, in aqueous solution at pH = 2, P4VP block was demonstrated to be the shell-forming chain of the polymer aggregates as temperature increased. The P4VP-shell started to dehydrate when PN4P was heated to 25–30 °C, along with a gradual transition of P4VP chains from coil to globule and a cluster size (*D*_h_) evolution from 338 to 350 nm (Fig. 10A). The more folded shell-forming chains may lead to the geometric confinement of 4 and may favor its RIR effect, making it more emissive (Fig. 10B). Remarkably intensive dehydration in the temperature range of 31 to 39 °C would result in the formation of more and more dense aggregates, as proven by the transition of the polymer cluster size from 360 to 803 nm from DLS data (Fig. 10A), which greatly triggered the RIR process of the 4 units and thus led to a 2.10-fold more striking emission increase of PN4P, revealing its AIE feature from 25 to 33 °C (Fig. 10B). At higher temperatures above the CST, the molecular motions including the vibration and intra-molecular rotation of the fluorescent group 4 would be spurred, which may gradually and mildly counterbalance the AIE effect and help to explain the observed slow emission decay above 33 °C (Fig. 9B and 10B). Meanwhile, similar thermal-responsive emission trends in water at pH = 7 and 11 also contributed to supporting the AIE feature of the probe. Thirdly, as illustrated in Section 3.2.2, PN4P could form core–shell micelles with a PNIPAM shell at pH > 4.0 or micelles with a P4VP shell at pH = 2, and the clustering degree of PN4P would become greater as temperature increases, as reflected in the DLS data shown in Fig. 10A. It seems that the P4VP shell is more easily dehydrated subject to temperature rise than PNIPAM as the shell due to the 2.43-fold enhancement in the *D*_h_ of PN4P clusters in water at pH = 2, whereas the respective counterpart was only increased by 1.10- (pH = 7) and 1.24-fold (pH = 11) as compared with that at 20 °C. The remarkable most intensive dehydration of the P4VP shell at pH = 2 as compared to that of the PNIPAM shell at pH = 7 and 11 would make PN4P the most strongly aggregated and most AIE-active in acidic water (Fig. 10), which supports the 2.10-fold most distinguished thermal-responsive emission enhancement in water at pH = 2 (Fig. 10B). Furthermore, the question arises as to why the FL at pH = 11 is always greater than the FL at pH = 7 at comparable temperatures ranging from 15 to 50 °C (Fig. 10B). As shown in Fig. 8A, the respective CST of PN4P is about 30 and 27 °C for water solutions at pH = 7 and 11, signifying the poorer solubility or stronger dehydratation of the shell-forming PNIPAM block in pH = 11 solution, which would result in a denser and larger polymer clustering size at pH = 11 with increasing temperature than that at pH = 7, as proven in Fig. 10A. The denser polymer aggregation at pH = 11 would induce a stronger RIR effect of the fluorogen 4 and make it more emissive than that at pH = 7, which again favors the AIE feature of PN4P. As for the deprotonation-driven emission decay mechanism, there primarily exists the strongly emissive PN4P-n at pH = 2 while under conditions of pH = 7 and 11, both the strongly emissive PN4P-n and less emissive PN4P-d will coexist. The shift of the deprotonation equilibrium towards more PN4P-d at pH = 7 and 11 would lead to a decrease in the amount of PN4P-n and a resultant FL decay, which could clarify why the FL (pH = 2) > FL (pH = 7 and 11) at constant temperature ranging from 15 to 50 °C (Fig. 10B).

In short, although the AIE and the deprotonation-driven emission decay may be the two major factors responsible for the dual responsive emission behavior of PN4P in water, there may exist negligible deprotonation and the strongest AIE effect due to the most intensive dehydration of the P4VP shell at pH = 2, whereas there is strong deprotonation and moderate AIE at pH = 11, and then weak AIE at pH = 7, owing to the poorer dehydration of the PNIPAM shell at pH > 4, which could explain why the FL (pH = 2) > FL (pH = 11) > FL (pH = 7) at constant temperature ranging from 15 to 50 °C (Fig. 10B). Additionally, an increase in the solution basicity would generally shift the deprotonation equilibrium of PN4P towards more PN4P-d, according to our previous work,^26^ but we actually observed that FL (pH = 11) > FL (pH = 7) at constant temperatures (Fig. 10B), which obviously indicates that the AIE effect plays a more important role than the deprotonation-driven decay mechanism.

**Fig. 11:**
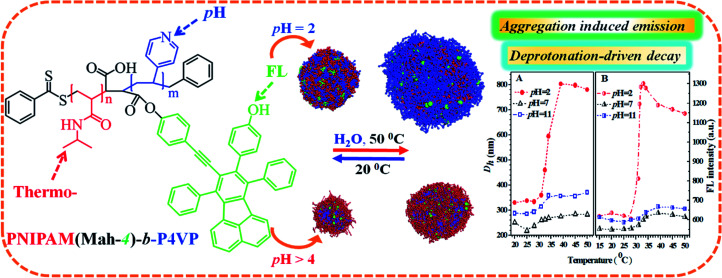
The proposed pH- and thermal-responsive micellar/clustering structures and emission mechanisms of the nanoprobe PN4P in water.

Based on the above data and discussion, we can propose a possible dual-responsive clustering/micellar structure and emission mechanism of the probe in water as displayed in Fig. 11.

### 3.3 Sensitivity and reversibility

**Fig. 12:**
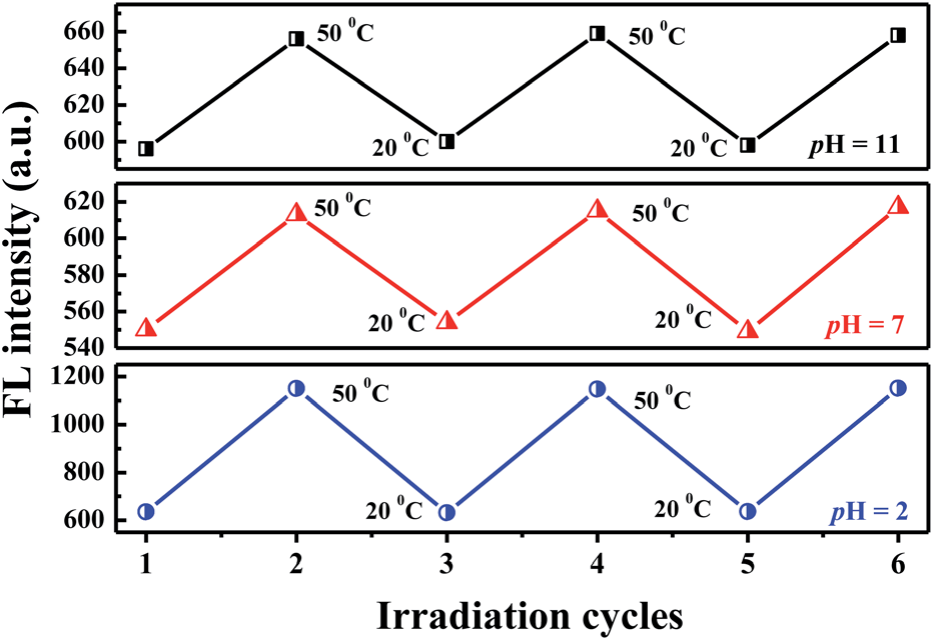
FL cycling between 20 °C and 50 °C of PN4P in water (*λ*_ex_ = 390 nm, 0.5 mg mL^−1^).

The variations in the FL peak intensity of PN4P in aqueous solutions at constant pH are displayed in Fig. 12, where the temperature was switched repeatedly between 20 and 50 °C. At constant pH values of 2, 7 and 11, the data visibly indicate that our PN4P probe can adjust its intensity reversibly by temperature tuning. The reason could be as follows. The shell-forming chains might have a reversible coil-to-globule phase transition against temperature at constant pH in aqueous solution, and therefore the corresponding emission of PN4P behaves reversibly with temperature switching. That is, at constant pH in aqueous solution, the phase transition of the copolymer would take place reversibly, despite the cooling/heating process, thus illustrating reversible emission decay/increase along with the temperature change. It was found that the emission peak intensity at 50 °C and pH = 2 was enhanced by about 1.81-fold as compared with that at 20 °C, whereas the counterpart was increased only 1.12- (pH = 7) and 1.10-fold (pH = 11) as compared with that at 20 °C. This may be attributed to the strongest dehydration of P4VP as the micellar shell at pH = 2 being subject to an increase in temperature as compared to PNIPAM as the shell at pH = 7 and 11. The most intensive dehydration of the P4VP shell at pH = 2 would make PN4P the most strongly aggregated and the most AIE-active in acid water, favoring the strongest emission enhancement at pH = 2.

Regarding the proper use of the probe, the pH responses based on the absorption data shown in Fig. 8B were employed only to clarify the critical pH value of the probe. We read the pH response *via* the emission data of the probe since the emission data are more sensitive to pH changes as shown in Fig. 7C, which shows a monotonic variation in emission intensity against pH within the pH range of 2 to 7, indicating that PN4P is a good pH sensor within the pH range of 2 to 7.

Combined with the data shown in Fig. 7C, Fig. 10B and 12, we are sure that the probe shows the strongest sensitivity at pH = 2, then at pH = 7 and the weakest sensitivity at pH = 11. The probe could, therefore, be a promising sensor that is suitable for working within the pH range of 2 to 7 (Fig. 7C) and the temperature range from 28 to 40 °C (Fig. 10B), simultaneously with very strong pH sensitivity, especially in the pH range of 2–4 (Fig. 7C).

## 4. Conclusions

We developed a new fluorescent pH- and thermo-meter, PN4P, in this work. The micellar/clustering structures of the probe in water were investigated by solution dispersibility, NMR, TEM, DLS, FL and UV-vis measurements, and the emission performance was systematically examined by temperature and pH tuning.

We illustrated the plausible dual-responsive micellar structures and emission mechanisms of the probe in water. The critical solution temperature of the probe in water is about 32 °C (pH = 2), 30 (pH = 7) and 27 °C (pH = 11). The critical pH value of the probe in aqueous solution was found at about 4, and the micelles showed a core–shell inversion in response to pH and thermal stimuli accompanied by remarkable emission tunability. Acidochromic changes in the emission band from 450 (pH > 4) to 490 nm (pH = 2) was observed in water, and the probe showed the best sensitivity at pH = 2. PN4P demonstrates obvious deprotonation-driven emission decay and AIE features in different microenvironments and displays considerable temperature and pH responses, as well as good reversibility. The probe is more sensitive within the pH range of 2 to 7, and the temperature range from 28 to 40 °C. Therefore, we see that our probe is suitable for working in a temperature and pH range that is interesting for biomedical applications, and therefore, we can expect that our PN4P probe is suitable for use as a biosensor or in the biomedical field, due to the suitable temperature and pH responsive range.

## Conflicts of interest

There are no conflicts of interest to declare.

## Supplementary Material
